# IQGAP1 is an oncogenic target in canine melanoma

**DOI:** 10.1371/journal.pone.0176370

**Published:** 2017-04-26

**Authors:** Becky H. Lee, Poornima H. Neela, Michael S. Kent, Ashley M. Zehnder

**Affiliations:** 1 School of Veterinary Medicine, University of California-Davis, Davis, California, United States of America; 2 Program in Epithelial Biology, Stanford University, Stanford, California, United States of America; 3 Department of Surgical and Radiological Sciences, School of Veterinary Medicine, University of California-Davis, Davis, California, United States of America; Rutgers University, UNITED STATES

## Abstract

Canine oral mucosal melanoma is an aggressive malignant neoplasm and is characterized by local infiltration and a high metastatic potential. The disease progression is similar to that of human oral melanomas. Whereas human cutaneous melanoma is primarily driven by activating mutations in Braf (60%) or Nras (20%), human mucosal melanoma harbors these mutations much less frequently. This makes therapeutic targeting and research modeling of the oral form potentially different from that of the cutaneous form in humans. Similarly, research has found only rare Nras mutations and no activating Braf mutations in canine oral melanomas, but they are still reliant on MAPK signaling. IQGAP1 is a signaling scaffold that regulates oncogenic ERK1/2 MAPK signaling in human Ras- and Raf- driven cancers, including melanomas. To investigate whether IQGAP1 is a potential target in canine melanoma, we examined the expression and localization of IQGAP1 in primary canine melanomas and canine oral melanoma cell lines obtained from the University of California-Davis. Using CRISPR/Cas9 knockout of IQGAP1, we examined effects on downstream ERK1/2 pathway activity and assayed proliferation of cell lines when treated with a peptide that blocks the interaction between IQGAP1 and ERK1/2. We observed that canine IQGAP1 is expressed and localizes to a similar extent in both human and canine melanoma by qPCR, Western blot, and immunofluorescence. Deletion of IQGAP1 reduces MAPK pathway activation in cell lines, similar to effects seen in human Braf^V600E^ cell lines. Additionally, we demonstrated reduced proliferation when these cells are treated with a blocking peptide in vitro.

## Introduction

Using naturally occurring models of animal disease to study complex diseases, such as cancer, allows researchers to better translate their findings, as genetically engineered mouse models have severe limitations when attempting to translate therapy to human patients[1]. Canine mucosal melanoma offers a unique model to study responses to signaling and therapeutics[2]. It is similar to human mucosal melanomas in that activating Braf and Nras mutations are very rare[3–5], in contrast to human cutaneous melanoma. Furthermore, canine oral melanomas are dependent on AKT and MAPK signaling[6, 7] and have altered expression of tyrosinase, p16, Pten and P53 similar to what is observed in human melanomas[8, 9]. The clinical progression of canine oral melanomas is also similar to that of human oral mucosal melanomas as both are locally invasive and often metastasize through the lymphatic system. The prognosis of oral melanomas in humans and dogs is poor because it often recurs even with multimodal therapy including surgery, radiation, and chemotherapy[10].

**Fig 1:**
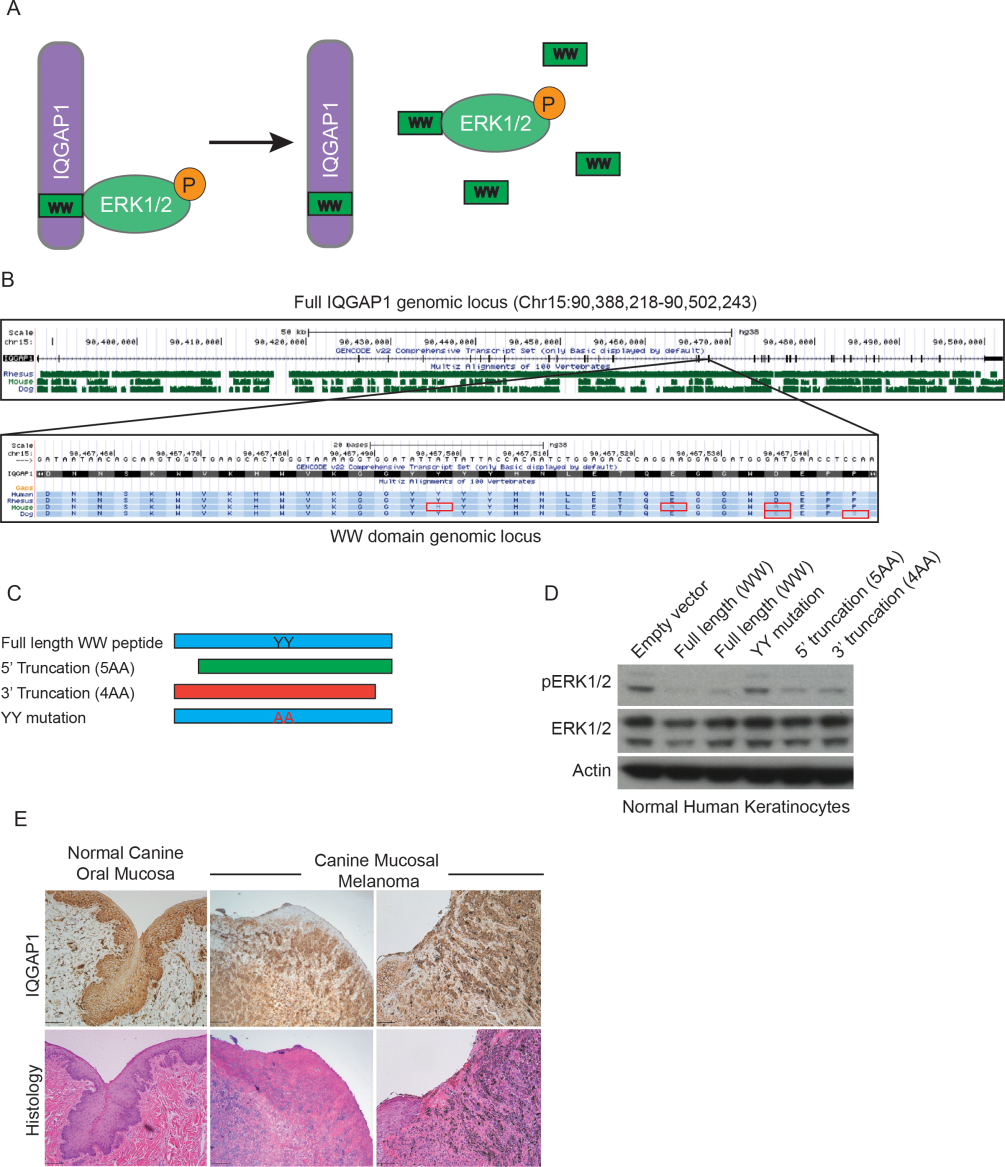
Canine IQGAP1 is more highly conserved than mouse IQGAP1, particularly within the WW domain. (A) Schematic of function of WW peptide interruption of binding between IQGAP1 and ERK1/2. (B) IQGAP1 genomic locus with mouse and canine conservation tracks. Specific amino acid alignments of the WW domain [inset]. (C) Diagram of WW peptide mutations. (D) Immunoblot of primary human keratinocytes infected with either the full-length WW domain, one with two mutated central YY residues, a 5’ truncation missing 5AA and a 3’ truncation missing 3AA. (E) Histology and IQGAP1 immunohistochemistry from normal canine oral mucosa and primary canine mucosal melanoma. Scale bar = 100μm.

IQGAP1 is a signaling scaffold that regulates oncogenic ERK1/2 MAPK signaling in human cancer[11, 12]. We recently described a new therapeutic strategy for Ras/MAPK-driven cancers, which does not target the core pathway members, but instead targets the interaction between IQGAP1 and ERK1/2. This therapeutic strategy inhibits cancer progression in a number of in vitro and in vivo models of cancer[11]. That research demonstrated therapeutic efficacy either with genetic depletion of IQGAP1 or by an interfering peptide derived from the IQGAP1 WW domain (Fig 1A) that disrupted association of ERK1/2 with full-length IQGAP1[11]. Targeting IQGAP1 reduces ERK1/2 pathway activity in neoplastic cells while preserving normal ERK1/2 MAPK function in physiologically normal cells, demonstrating a tumor-specific efficacy. More recently, additional investigators have reported the usefulness of targeting kinase scaffolds in pancreatic cancer[13, 14] and melanoma[15], confirming that scaffold targeting may represent a promising new general approach to cancer treatment. Our previous work investigated IQGAP1 targeting in human and mouse models, but not in canine spontaneous cancer models. This manuscript expands the clinical translation of scaffold targeting therapies into a clinical relevant model of mucosal melanoma by examining the expression of IQGAP1, the effects on proliferation as well as modulation of the ERK1/2 MAPK pathway in canine melanoma where IQGAP1 is deleted or targeted therapeutically.

## Materials and methods

### Cell line culture

Human cancer cell lines were maintained in DMEM (SK-Mel28, SK-Mel5, WM2664, A375) or RPMI (Colo829) (Gibco) supplemented with 10% FBS according to recommendations from ATCC. Canine melanoma cell lines CMM3 and CMM5 were obtained from one of the author’s laboratory (MSK), from where they were derived, have been validated to be of canine origin with no inter-species contamination (IDEXX), have been tested to be free of mycoplasma contamination (AZ) and were immunologically typed as being positive for the following melanoma specific markers: Melan-A, S-100, MITF and Tyrosinase[16]. They were maintained in RPMI (Gibco) supplemented with 10% FBS. Primary human epidermal keratinocytes were isolated from discarded neonatal surgical specimens and cultured as previously described[17].

### Drug treatments and inhibitors

GSK1120212 (Trametinib) was purchased from SelleckChem (#S2673) and resuspended in DMSO as per manufacturer recommendations to a stock of 10mM. WW and Scr peptide were obtained from CS Bio Co., manufactured as described previously and reconstituted to a stock of 2.5mM in water[11].

### Canine melanoma samples

Four primary canine mucosal melanomas and two samples of normal canine oral mucosa were obtained from biopsy tissue collected under IACUC approved protocol 18749 at the University of California Davis School of Veterinary Medicine.

### Quantitative RT-PCR (qRT-PCR)

RNA was harvested from melanoma cell lines by treatment using the RNeasy kit (Qiagen). The following primers were used for this study:

Human 18s: forward, 5’-GCAATTATTCCCCATGAACG-3’ and reverse, 5’- GGCCTCACTAAACCATCCAA-3’;
IQGAP1: forward, 5′-TTCGCCACTACCCAGACCTTGTTT-3′ and reverse, 5′-CCTGTCTTGGATGTGGCCTTTGG-3′;
Canine HPRT: forward, 5’- AAGGACCCCTCGAAGTGTTG-3’, reverse 5’- ACTAAGCAGATGGCCACAGAA-3’
Canine IQGAP1: forward, 5’- AGAAGTGGCCCAGCATTACC-3’, reverse 5’- CAGCTGACTCGTCCTGTGTT-3’

RT-PCR conditions: 95°C for 5min, 45 cycles of 95°C for 10s, 58°C for 30s, 72°C for 30s. RT-PCR was performed on a Roche Lightcycler 480.

### IQGAP1 CRISPR

For CRISPR-Cas9 infections, cells were infected with PLenti-CRISPR virus with four sgRNAs targeting IQGAP1. sgRNA sequences were based on published sequences for human IQGAP1 sgRNAs[18] and altered to match the equivalent canine sequence based on sequence alignments. Cells were selected for 3 days in puromycin and tested by immunoblot for loss of IQGAP1 protein expression.

sgRNA guide sequences:

IQ1: AGGACATTCGGAATCAGCGG
IQ4: TGGTTGGATGAAATTCAAGG
IQ5: GATGGAGTCTCAGACGGGAGA
IQ7: GATGGAGTCTCAGACGGGAGA

### Protein expression analysis

Immunoblots were performed with the following antibodies: rabbit antibody to ERK1/2 (1:1,000, Cell Signaling, 9102), rabbit antibody to pERK1/2 (Thr202/Tyr204; 1:1,000, Cell Signaling, 9101), rabbit antibody to IQGAP1 (1:500, Abcam, 86064), rabbit antibody to RSK1 (1:1000, Cell Signaling 9333), rabbit monoclonal antibody to phospho-p90RSK (1:1000, Cell Signaling 8753), and mouse monoclonal antibody to β-actin (1:10,000, Sigma, A1978).

### Immunofluorescence

Cell lines were grown on chamber slides, fixed and permeabilized by incubation 4% paraformaldehyde for 10 minutes followed by blocking in 10% horse serum in PBS for 1 hour. Tissue sections were then incubated with primary antibodies, followed by Alexafluor-488 conjugated goat anti-mouse secondary antibodies (Molecular Probes). The following antibodies were used: IQGAP1 (1:100, Santa Cruz sc-376021). Tissue was counterstained with Hoescht to visualize cell nuclei.

### Immunohistochemistry

Immunohistochemistry was performed as previously described[19] with mouse antibody to IQGAP1 (1:100, Santa Cruz sc-376021). Secondary staining with biotinylated horse secondary to mouse IgG at 1:1000 for 1hour (Vector Laboratories). Negative control samples were stained using the same protocol without addition of primary antibody. Antibody was tested for specificity using cell lines with IQGAP1 depletion using CRISPR-Cas9 as described above.

### Cell viability assay

Cancer cell lines were seeded in equivalent, low-density cultures in duplicate in 24-well plates. Twenty-four hours after seeding, the medium was removed and replaced with 500μl of a 5:1 mixture of cell medium and cell titer blue reagent (Promega). Cultures were incubated with this mixture for 2h at 37°C in the dark. Fluorescence (560nm/590nm (emission/excitation) for 100 μl in triplicates for each sample was recorded. Values plotted correspond to readings taken while control samples were still growing exponentially. For IC50 assays, cells were seeded into 96-well plates. Twenty-four hours after seeding, serial dilutions of GSK1120212 were prepared in DMSO and added to cells, yielding final drug concentrations ranging from 0 to 30μM. Cells were treated daily and incubated for 72h after the addition of drug. Data from growth-inhibition assays were modeled using a nonlinear regression curve fit with a sigmoid dose response. Cell proliferation curves were generated graphically as % control +/- SEM using the following formula:

(A = absorbance; TREAT = treatment group; CON = control or untreated; ini = initial; fin = final)
Percent control growth: 100% * (ATREAT − ACONini) / (ACONfin − ACONini)
Percent standard error of the mean: 100% * (SEMTREAT/(ACONfin − ACONini)),

where the SEM = (SDEV/ n ^ 0.5), n = number of replicates).

Half-maximal inhibitory concentration (IC_50_) values were generated using Prism 5 (GraphPad).

### Statistics

Each data panel is representative of at least three independent replicate experiments or biological replicates using different cell lines. Analysis of statistical significance for the proliferation assays was performed using Student's *t* tests. A p-value of < 0.05 was considered statistically significant.

## Results

### Canine IQGAP1 is more highly conserved than mouse IQGAP1, particularly within the WW domain

Examination of the canine IQGAP1 locus reveals many similarities to human IQGAP1, particularly within the portion that has been targeted as a cancer therapeutic, the WW region (Fig 1B–lower panel). This contrasts with the degree of dissimilarity between human and mouse IQGAP1 evident in the upper UCSC track and in the WW domain highlighted in red (Fig 1B–upper and lower panel). Within this 33-amino acid region, there are only 2 amino acids that differ between the two species (29D and 32P). Previous experiments performed in normal human keratinocytes during initial peptide testing determined that these C-terminal amino acids of the WW domain are not necessary for the peptide mimic to reduce ERK1/2 phosphorylation (Fig 1C and 1D). This is compared to mutation of two central YY residues, which are necessary for the reduction of ERK1/2 phosphorylation, as observed by alanine substitution at these residues. This indicates the 2 amino acid difference between the human and canine IQGAP1 sequence is most likely not required for the specific interaction with ERK1/2. With the observation of the largely conserved IQGAP1 sequence, we hypothesized that targeting IQGAP1 in the canine melanoma model should be an effective therapy in controlling neoplastic cell populations.

Using spontaneously occurring primary canine mucosal melanoma samples (n = 4) from previously non-treated animals and normal canine mucosal samples for comparison (n = 2) we performed IQGAP1 immunohistochemistry (Fig 1E). Negative control for IQGAP1 immunohistochemistry is shown in S1 Fig. These studies reveal a similar expression pattern to what is observed in multiple human cancer types[20–23]. Staining is primarily cytoplasmic and diffuse, with more prominent IQGAP1 staining at the periphery of tumors.

### Canine melanoma lines are dependent on ERK1/2 and express IQGAP1 similar to human Braf^V600E^ melanoma lines

**Fig 2:**
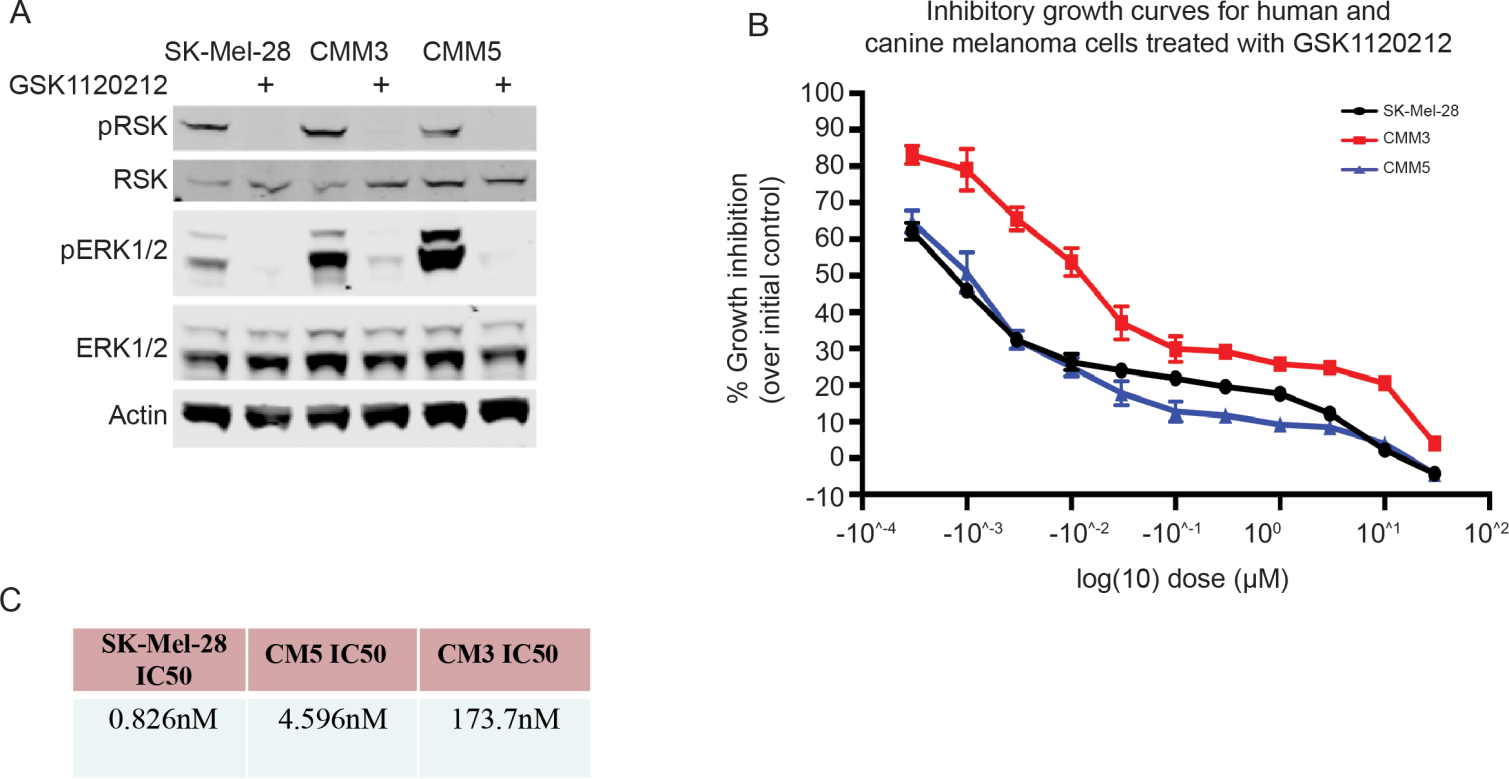
Canine melanoma lines are dependent on ERK1/2 similar to human Braf^V600E^ melanoma lines. (A) Immunoblot for human Braf^V600E^ melanoma cells (SK28) and two canine mucosal melanoma cell lines (CMM3, CMM5) treated with DMSO or GSK1120212 (Trametinib) for 8hrs. (B) Growth curves for two canine mucosal melanoma cell lines (CMM3, CMM5) and human Braf^V600E^ melanoma cells (SK-mel-28) treated with GSK1120212 ranging from 0.0001μM to 30μM. Values represent 3 biological replicates for CMM lines and 2 biological replicates from SK-Mel-28. (C) IC50 values from 4-day proliferation assays in cells as in (A).

We initially determined that two canine melanoma cell lines responded to ERK1/2 MAPK inhibition similarly to human melanoma cell lines using GSK1120212, a specific MEK1/2 kinase inhibitor that is currently being tested in human cancer clinical trials[24]. These studies confirm that the canine melanoma cell lines used for our preliminary trials respond strongly to ERK1/2 MAPK inhibition with a reduction in pERK1/2 as well as pRSK, a canonical substrate of ERK1/2 (Fig 2A). Growth inhibition curves for GSK1120212 treatment in two canine melanoma lines (CMM3 and CMM5) as well as for a human Braf^V600E^ mutated melanoma line (SK-Mel-28) for comparison are presented in Fig 2B. We also calculated IC50s for all of these lines using a dosing curve over 3 days of drug treatment. The IC50 for SK28 agrees with what is published for known sensitive lines[25]. CMM5 is also considered sensitive with a 4.5 nM IC50. CMM3 is less so with an IC50 of 173nM, but still does respond to treatment. Growth inhibition studies performed in triplicate provide estimates of IC50 for this MEK inhibitor are in the range of what are considered “sensitive” cells, although there is a distinct difference between the canine lines (Fig 2C). For additional mechanistic studies, we focused on CMM5 since it was more responsive to ERK1/2 MAPK inhibition.

### Canine melanoma lines express IQGAP1 similar to human Braf^V600E^ melanoma lines

**Fig 3:**
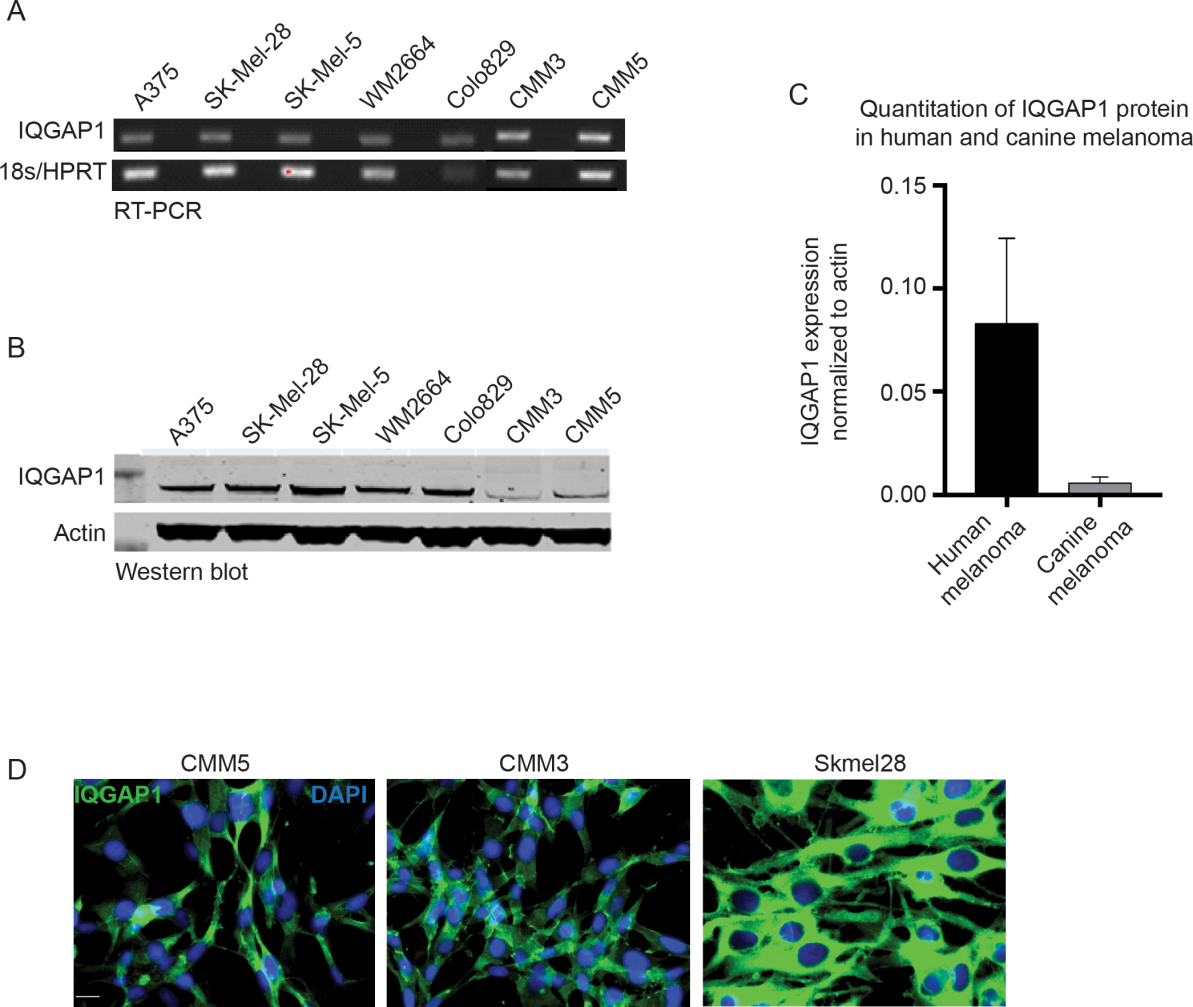
Canine Melanoma lines express IQGAP1 similar to human Braf^V600E^ melanoma lines. (A) Gel electrophoresis (top) from IQGAP1 RT-qPCR for five human Braf^V600E^ melanoma cell lines and and two canine mucosal melanoma cell lines (CMM3, CMM5). (B) IQGAP1 immunoblot from cell lines as for the qPCR. (C) Quantitation of human and canine IQGAP1 protein expression normalized to actin from western blot in (B). (D) Immunofluorescence for IQGAP1 in human Braf^V600E^ melanoma cells (Skmel28) and two canine mucosal melanoma cell lines (CMM3, CMM5). Scale bar = 50μm.

We compared the RNA and protein expression of canine and human IQGAP1 in our cell lines compared to a panel of human Braf^V600E^ melanoma cell lines. Fig 3A shows a DNA gel showing results from RT-qPCR amplifying IQGAP1 in human and canine cell lines. Housekeeping genes (18s for human melanoma lines and HPRT for canine melanoma lines) are shown below for normalization and the Ct values for IQGAP1 are provided in S2A Fig along with the value for the housekeeping genes. RT-qPCR curves are presented in S2B Fig. The canine cell lines appear to express IQGAP1 transcripts slightly more than the human cell lines. However, when expression is examined by western blot (Fig 3B and 3C), the protein expression appears lower. This may be due to differential protein expression or it may be that the IQGAP1 antibody does not recognize the canine form with the same sensitivity. When we examine IQGAP1 localization by immunofluorescence (Fig 3D), we see a similar cytoplasmic staining pattern for the canine cells and the human melanoma line SK-Mel-28.

### IQGAP1 deletion or interruption of IQGAP1-ERK1/2 interaction reduces ERK1/2 pathway signaling and proliferation in canine melanoma lines

**Fig 4:**
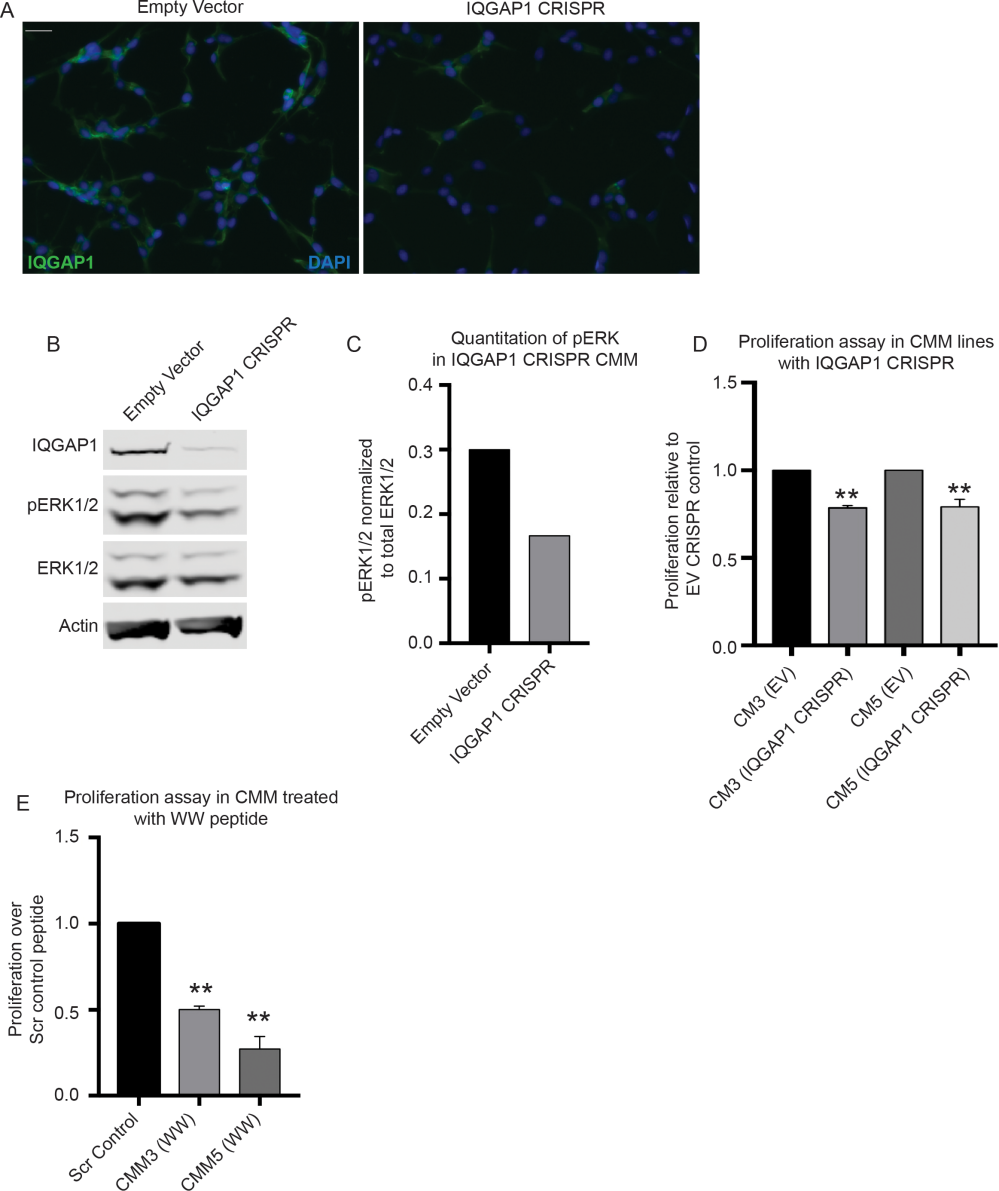
IQGAP1 deletion or interruption of IQGAP1-ERK1/2 interaction reduces ERK1/2 pathway signaling and proliferation in canine melanoma lines. (A) IQGAP1 Immunofluorescence in CMM5 melanoma cell line treated with either EV or IQGAP1 targeting sgRNA. Scale bar = 50μm. (B) Immunoblot for pERK1/2 and IQGAP1 in cells from (A). (C) Quantitation of human and canine pERK1/2 protein expression in IQGAP1 CRISPR canine melanoma cells normalized to ERK1/2 from western blot in (B). (D) Six-day proliferation assays in CMM3 or CMM5 with IQGAP1-targeting CRISPR, ** p-val <0.01. (E) Six-day proliferation assays in CMM5 (left) or CMM3 (right) cells treated with either Scr or WW peptide, ** p-val <0.01.

We next examined ERK1/2 phosphorylation in the context of IQGAP1 depletion in the canine melanoma line that was most sensitive to MEK-inhibition (CMM5). We utilized Cas9-CRISPR targeting IQGAP1 and one of the same sgRNAs we used for the human lines as the target sequence was identical. Immunofluoresence studies indicated that there is a reduction in IQGAP1 protein expression (Fig 4A). The protein is also substantially reduced on immunoblot (Fig 4B). When pERK1/2 is quantified on immunoblot and normalized to ERK1/2, we observed an approximately 50% reduction in pERK1/2 in cells with IQGAP1 depletion, indicating a reduction in ERK1/2 pathway activation (Fig 4C).

We assessed proliferation in both canine melanoma cell lines with IQGAP1-targeting Cas9-CRISPR and note a consistent and statistically significant decrease compared to empty vector (EV) controls using four different sgRNAs in three biological replicates (Fig 4D). Finally, we evaluated proliferation in both canine melanoma lines treated with WW peptide over a 6-day time period. In both CMM5 and CMM3, we see a reduction in growth with the active peptide treated at 10μM compared to scrambled control peptide (Fig 4E).

## Discussion

This study explores the utility of expanding IQGAP1 targeted therapies to canine models of melanoma. The advantage of characterizing this novel therapeutic target in an alternative cancer model is the ability to better predict and characterize potential human responses to therapy compared to performing pre-clinical testing in conventional rodent models. Treating naturally occurring cancers better models the clinical condition of human patients.

For these studies, we use canine mucosal melanomas as these are the most common canine melanoma presentations and currently available cell lines are of mucosal origin. These tumors differ from human cutaneous melanomas in that Braf and Nras mutations are not frequently observed. This is similar to what is found in human mucosal melanomas as well. However, canine mucosal melanomas are reportedly sensitive to MAPK targeted therapies and have upregulated levels of pERK1/2, suggesting that these tumors are reliant on MAPK signaling for tumorigenesis. It is unknown if there are mutations elsewhere in the ERK1/2 MAPK pathway or if the increased pathway activation is primarily due to increased expression of pathway components.

We initially characterized the presence and localization of IQGAP1 in primary canine mucosal melanomas, observing that there does appear to be increased IQGAP1 expression, particularly co-localizing with melanocytes as well as at the tumor edge. We additionally examined the expression and localization of IQGAP1 in two canine mucosal melanoma cell lines. While there seems to be a modest difference in the absolute expression of IQGAP1 between human and canine melanoma cell lines (human cell lines express higher levels), the localization to the cytoplasm and the cell periphery is very similar based on immunofluorescence. It is important to note that the antibody used for the immunohistochemistry studies was initially validated in cell lines with IQGAP1 knockdown using CRISPR Cas9 to determine specificity.

Based on these observations, we then sought to confirm that the cell lines were sensitive to MAPK targeted therapies, as this has generally been a good predictor as to which lines will also respond to IQGAP1 targeted therapy. Western blot analysis, as well as IC50 curves, confirmed that the lines respond to MEK inhibition with a downregulation in pERK1/2 and a significant decrease in proliferation. The calculated IC50 values are higher than for a human melanoma cell line used here for comparison, but still within the range of sensitive lines. It is interesting to note that there is an almost 50-fold increase in IC50 between the human melanoma cell line and the more resistant of the two canine lines tested. Reported IC50s range from 0.48nM to 0.52nm for Braf^V600E^ mutant melanoma lines to up to 174nM for Kras mutant lines[25]. MAPK wild type lines are insensitive to MEK inhibition up to 10μM based on in-vitro testing (SelleckChem). The canine lines used for these experiments are within the range of MAPK mutated human cancer lines. It is known that these lines have wild type Braf, however, the Nras mutational status is unknown[26].

Using a pLenti CRISPR Cas9 system, we were able to successfully deplete the expression of IQGAP1 protein in a bulk population of cell line CMM5 (the more sensitive of the two lines tested). To the authors’ knowledge, this is the first time that CRISPR Cas9 has been used to delete genes in canine cancer cell lines. We confirmed knockdown using immunofluorescence as well as Western blot. The lines in these experiments still exhibit some protein expression because these are mixed cell populations and do not represent clonal populations. In these mixed population cells, we observed an approximately 50% reduction in pERK1/2 compared to WT cells, suggesting that we are, indeed, impacting ERK1/2 MAPK function. In CMM cells with IQGAP1 CRIPSR, we notice a consistent and statistically significant reduction in proliferation.

Finally, we performed proliferation assays to determine if treatment with the WW peptide would result in decreased proliferation in the canine lines. This appeared to be a viable treatment strategy based on the observation that the WW domain of IQGAP1 is the same between canines and humans except for two residues in the C-terminal end of the protein. We have demonstrated in human keratinocytes that the four C-terminal residues are not critical to reducing signaling through IQGAP1-ERK1/2. We noted a significant decrease in proliferation with the WW treated lines compared to Scr treated controls. Interestingly, we noted that WW peptide treatment has a greater absolute effect on proliferation combined with the partial knockout using IQGAP1 CRISPR. There may be several explanations for this observation, including incomplete deletion of IQGAP1 in this pooled CRISPR population, potential effects of IQGAP1 deletion on additional signaling pathways, effects WW peptide treatment on other ERK1/2 interactions or other “off-target” effects that have not been fully characterized.

These data together provide a preliminary justification for investigating the use of the WW peptide in canine mucosal melanomas, as a model of human disease and for the purpose of treating canine patients. The findings in this manuscript further support canine oral melanoma model as a relevant spontaneous research model for human melanomas.

## Supporting information

S1 Fig Negative control IQGAP1 immunohistochemistry. (A) Immunohistochemistry of a canine mucosal melanoma sample from same sections as in Fig 1D processed without primary IQGAP1 antibody. Scale bar = 200μm.
(TIF)

S2 Fig RT-qPCR amplification curves for IQGAP1 in human and canine melanoma. (A) Quantitation of the qPCR based on Ct values, with house-keeping genes for human and canine shown for comparison. (B) Amplification curves for two canine melanoma lines (CMM) and five human melanoma lines (HM). Housekeeping genes TBP and HRPT1 were used for the CMM lines and 18s was used for the HM lines.
(TIF)
