# Supplementary figures and images for: IQGAP1 is an oncogenic target in canine melanoma
Source: PLoS One. 2017 Apr 26;12(4):e0176370. doi: 10.1371/journal.pone.0176370 (PMC5406000; doi:10.1371/journal.pone.0176370)

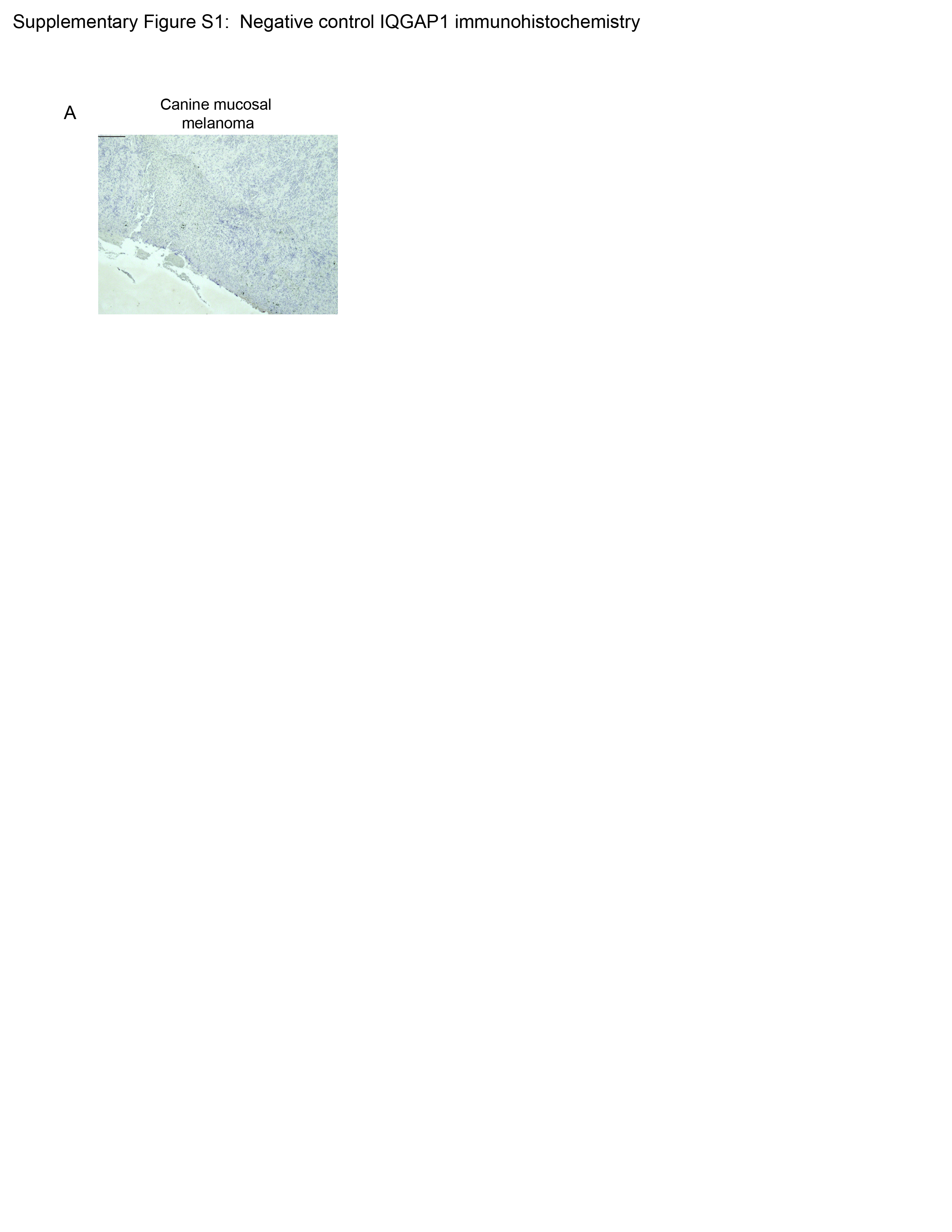

Supplement: S1 Fig — (A) Immunohistochemistry of a canine mucosal melanoma sample from same sections as in Fig 1D processed without primary IQGAP1 antibody. Scale bar = 200μm. (TIF) [file pone.0176370.s001.tif]

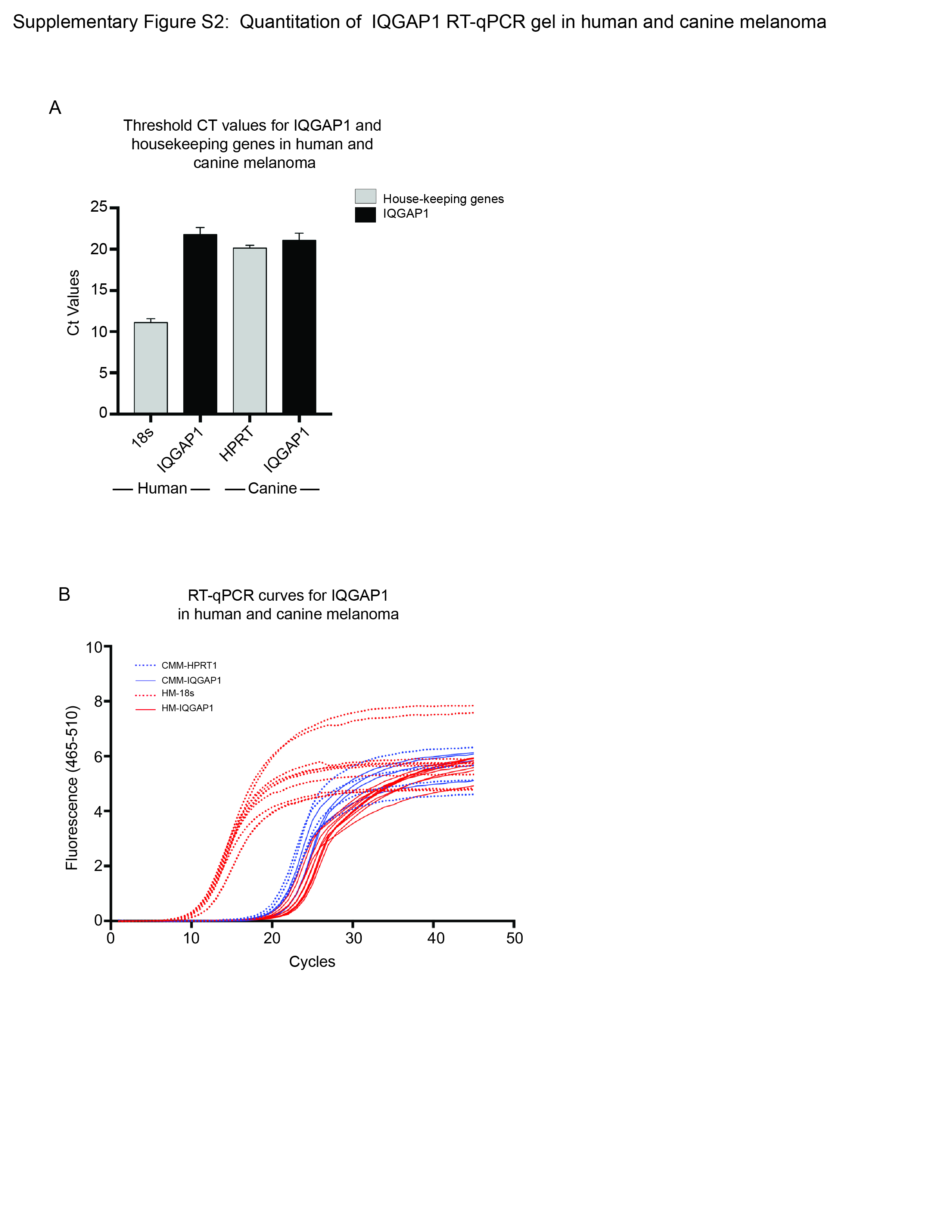

Supplement: S2 Fig — (A) Quantitation of the qPCR based on Ct values, with house-keeping genes for human and canine shown for comparison. (B) Amplification curves for two canine melanoma lines (CMM) and five human melanoma lines (HM). Housekeeping genes TBP and HRPT1 were used for the CMM lines and 18s was used for the HM lines. (TIF) [file pone.0176370.s002.tif]
